# Macrophage promotes fibroblast activation and kidney fibrosis by assembling a vitronectin-enriched microenvironment

**DOI:** 10.7150/thno.85250

**Published:** 2023-07-03

**Authors:** Yiling Peng, Li Li, Jingyue Shang, Haili Zhu, Jinlin Liao, Xue Hong, Fan Fan Hou, Haiyan Fu, Youhua Liu

**Affiliations:** 1 State Key Laboratory of Organ Failure Research, National Clinical Research Center of Kidney Disease, Division of Nephrology, Nanfang Hospital, Southern Medical University.; 2 Guangdong Provincial Institute of Nephrology, Guangzhou, China.

**Keywords:** Vitronectin, macrophage, fibroblast activation, kidney fibrosis, integrin αvβ5, Src signaling, ECM scaffold

## Abstract

**Background**: Renal infiltration of inflammatory cells including macrophages is a crucial event in kidney fibrogenesis. However, how macrophage regulates fibroblast activation in the fibrotic kidney remains elusive. In this study, we show that macrophages promoted fibroblast activation by assembling a vitronectin (Vtn)-enriched, extracellular microenvironment.

**Methods**: We prepared decellularized kidney tissue scaffold (KTS) from normal and fibrotic kidney after unilateral ischemia-reperfusion injury (UIRI) and carried out an unbiased quantitative proteomics analysis. NRK-49F cells were seeded on macrophage-derived extracellular matrix (ECM) scaffold. Genetic Vtn knockout (Vtn-/-) mice and chronic kidney disease (CKD) model with overexpression of Vtn were used to corroborate a role of Vtn/integrin αvβ5/Src in kidney fibrosis.

**Results**: Vtn was identified as one of the most upregulated proteins in the decellularized kidney tissue scaffold from fibrotic kidney by mass spectrometry. Furthermore, Vtn was upregulated in the kidney of mouse models of CKD and primarily expressed and secreted by activated macrophages. Urinary Vtn levels were elevated in CKD patients and inversely correlated with kidney function. Genetic ablation or knockdown of Vtn protected mice from developing kidney fibrosis after injury. Conversely, overexpression of Vtn exacerbated renal fibrotic lesions and aggravated renal insufficiency. We found that macrophage-derived, Vtn-enriched extracellular matrix scaffold promoted fibroblast activation and proliferation. In vitro, Vtn triggered fibroblast activation by stimulating integrin αvβ5 and Src kinase signaling. Either blockade of αvβ5 with neutralizing antibody or pharmacological inhibition of Src by Saracatinib abolished Vtn-induced fibroblast activation. Moreover, Saracatinib dose-dependently ameliorated Vtn-induced kidney fibrosis in vivo. These results demonstrate that macrophage induces fibroblast activation by assembling a Vtn-enriched extracellular microenvironment, which triggers integrin αvβ5 and Src kinase signaling.

**Conclusion**: Our findings uncover a novel mechanism by which macrophages contribute to kidney fibrosis via assembling a Vtn-enriched extracellular niche and suggest that disrupting fibrogenic microenvironment could be a therapeutic strategy for fibrotic CKD.

## Introduction

Chronic kidney disease (CKD) is a clinical syndrome defined as a persistent decline in kidney function. CKD carries a high risk of developing end-stage renal failure, and it lacks curative therapeutics for this devastating condition 1, 2. The prevalence of CKD in various regions ranges from 7% to 12% worldwide, and the number of people suffer from CKD surpasses 750 million worldwide 3, 4. Kidney fibrosis is the common pathological consequence of CKD, and its uncontrolled progression is often linked to kidney failure, regardless of the initial etiologies 5-7. However, our current therapeutic options for CKD are limited and often ineffective, largely due to an insufficient understanding of its underlying pathomechanism.

Infiltration of inflammatory cells including macrophages is a crucial event after kidney damage, which is usually accompanied by, and often precedes, renal fibrogenesis 8, 9. Among different kinds of inflammatory cells, macrophages are particularly interesting, as they are highly heterogeneous cell populations with distinct phenotypes and functions 10, 11. In response to kidney injury, macrophages are activated and polarized in a dynamic fashion, and display a spectrum of phenotypes ranging from pro-inflammatory (M1) to anti-inflammatory (M2) 12, 13. Although it is well documented that renal infiltration of macrophages is closely associated with the severity of kidney fibrosis 13, 14, exactly how macrophages contribute to fibroblast activation and expansion in the fibrotic kidney remains poorly understood.

Renal fibrotic damages are often unevenly spread across kidney parenchyma, indicating that local tissue microenvironment is of great importance in the initiation and progression of renal fibrosis 15. We recently put forward a hypothesis of the fibrogenic niche, a term coined to depict the unique profibrotic microenvironment that facilitates fibroblast activation 15, 16. It is conceivable that a string of cellular events take place in the fibrogenic niche, which include macrophage infiltration and fibroblast activation. However, whether macrophages contribute to the formation of the fibrogenic niche, thereby leading to fibroblast activation, remains to be determined.

In an effort to characterize the extracellular matrix (ECM) proteins, the framework and backbone of the fibrogenic niche 15, 17, 18, we performed the mass spectrometry analyses of the decellularized kidney tissue scaffold (KTS) from normal and CKD kidneys 19. We identified vitronectin (Vtn) as one of the most upregulated ECM proteins in the KTS of fibrotic kidney. Vtn is a multifunctional glycoprotein present in blood and the ECM compartment and plays an important role in various biological processes, such as cell adhesion, migration and invasion, proliferation and tissue remodeling 20, 21. Once released into the extracellular space, Vtn can interact with several proteins including cell membrane integrin receptors and transmits its signal to the target cells 22. A previous study reported that a high Vtn expression was detected in unilateral ureteral obstruction (UUO) model 23, and increased urinary Vtn was observed in kidney-transplanted patients 24. Furthermore, increased levels of Vtn are documented in models of tissue fibrosis of various organs such as liver and lung, as well as in degenerative skin or nervous system disease 25-27. These findings suggest that Vtn may play a role in orchestrating the formation of the fibrogenic niche in various organ disorders.

In this study, we report that Vtn is induced primarily in macrophages and targets interstitial fibroblasts in fibrotic kidney. Genetic knockout or knockdown of Vtn inhibits fibroblast activation and ameliorates renal fibrotic lesions, while overexpression of Vtn aggravates kidney lesions. Our findings suggest that macrophages contribute to fibroblast activation and kidney fibrosis by assembling a Vtn-enriched extracellular microenvironment.

## Methods

### Animal models

Unilateral ischemia-reperfusion injury (UIRI) and UUO were performed as described previously 16, 28. Vtn knockout mice were obtained from Cyagen Biosciences (stock no. KOCMP-22370-Vtn-B6N-VA). Identification of Vtn knockout mouse was accomplished by PCR 29. Hydrodynamic-based gene delivery assay was performed to knockdown or overexpression of Vtn in vivo, as previously described 19, 30. Mouse Vtn-shRNA plasmid (pLVX-Vtn-shR) was constructed by ligating Vtn siRNA sequences (5'-GGUUUCUCUGGCUGACCAATT-3') into the shRNA expression plasmid (pLVX-shRNA). Four days after UIRI, the mice were injected with either pLVX-Vtn-shR or control (pLVX-control) plasmids via tail-vein injection. For pharmacologic inhibition of Src signaling, mice were received a daily intraperitoneal injection of vehicle or Saracatinib at 20 mg/kg or 40 mg/kg body weight, beginning from day 4 post-surgery.

All animals were obtained from the Southern Medical University Animal Center (Guangzhou, China). All animal studies were performed by adherence to the NIH Guide for the Care and Use of Laboratory Animals and approved by the Experimental Animal Ethics Committee at the Nanfang Hospital, Southern Medical University.

### Human kidney biopsies and urine samples

We obtained kidney biopsies and urine samples from patients with CKD in the Nanfang Hospital, with written informed consent. Demographic and clinical information of the patients are shown in Table S1. Research involving human samples has been approved by the Human Subject Ethics Committee of the Nanfang Hospital, Sothern Medical University (NFEC-2021-051).

### Preparation of kidney tissue scaffold (KTS)

KTS was prepared as previously reported 16, 19. Briefly, each kidney was divided into four pieces along the sagittal plane through the blade. Kidney sections were decellularized using chemical reagents according to established procedures.

### Mass spectrometry proteomics analysis of the KTS

Proteome profiling of decellularized kidney tissues in sham and UIRI was described in our previous publication 19.

### Western blot analysis

The detail of Western blot procedures was described in previous report 16. The primary antibodies used are listed and presented in Table S2. Briefly, homogenates from mouse kidneys and cells were prepared with RIPA buffer. BCA assay was used to measure the concentration of total protein (Cat. ab287853, Abcam). Protein samples were reduced, denatured, subjected to Western blot analysis. The protein bands detection was performed using SuperEnhanced chemiluminescence detection reagents and visualization performed using Kodak X-ray film. Relative protein levels were analyzed by ImageJ software. All target proteins were normalized to an appropriate loading control (GAPDH or α-tubulin).

### Histology and immunohistochemical staining

Human and mouse tissues were fixed in 4% formalin overnight. The fixed tissues were dehydrated through an automatic dehydrator and 3-μm thick paraffin sections were prepared using conventional methods. Masson's trichrome staining (MTS) was performed using standard procedures and 3-µm sections were stained with MTS reagent. Immunohistochemical staining of human and mouse kidney tissues were conducted as previously described 16. Antibodies used are listed in Table S2.

### The enzyme-linked immunosorbent assay (ELISA) of Vtn

Human Vtn ELISA kit was purchased from Abcam (cat. ab234577). Human urinary Vtn levels were measured according to the manual instructions and corrected with urinary creatinine.

### Cell culture and treatment

Normal rat kidney interstitial fibroblasts (NRK-49F) and mouse monocyte/macrophage cell line (Raw264.7) were purchased from the American Type Culture Collection (ATCC) (Manassas, VA). Human proximal tubular epithelial cells (HKC-8) were provided by Dr. Lorraine C. Racusen (Johns Hopkins University, Baltimore, MD). NRK-49F, HKC-8, and Raw264.7 cells were serum-deprived for 24 h and incubated with recombinant human TGF-β1 at the concentration of 2 ng/ml. Some experiments were performed on NRK-49F cells pre-incubated with an integrin αvβ5-blocking antibody or the Src inhibitor Saracatinib for 1 h and then incubated in the absence or presence of Vtn.

### Preparation of macrophage-derived ECM scaffold

The decellularization of Raw264.7 cells was carried out as reported elsewhere 16. The TGF-β1-stimulated Raw264.7 were shaken for 120 min at 4 °C in calcium-free phosphate-buffered saline (PBS) containing EGTA (0.5 mM, pH 7.4) (#E3889; Sigma-Aldrich). We repeated the treatment 3-4 times until all cells are removed from the extracellular matrix. The ECM prepared from macrophages were washed with PBS and stored in a 4°C refrigerator for subsequent experiments.

### EdU-based cell proliferation assay

Detection of NRK-49F cell proliferation using the Click-iT Plus EdU Alexa Fluor 488 Imaging Kit (ThermoFisher Scientific). The cells were incubated with 10 µM EdU (5-ethynyl-2´-deoxyuridine) for 4 h before fixation and permeabilization. Cells and nuclei were stained with EdU cocktail solution and Hoechst 33342, respectively. Images were taken using fluorescence microscopy, and positive cells were counted.

### MTT assay

Cell mass was determined by the MTT [3-(4,5-dimethylthiazol-2-yl)-2,5-diphenyltetrazolium bromide] method. After the experiment, MTT (0.5 mg/ml) was added to the NRK-49F cells. After incubation for 4 hours, tetrazolium was released with dimethyl sulfoxide and the optical density was measured at 570 nm using an ELx800 Absorbance Reader (BioTek Instruments, Winooski, VT).

### Cell cycle analysis

Cell cycle analysis was performed using the cell cycle staining solution (Cat. CCS012; MultiSciences Biotech Co., Hangzhou, China). The DNA content was analyzed by a flow cytometer. The results were analyzed using the cell cycle analysis software ModFit LT (Verity Software House Inc., Topsham, ME).

### Serum creatinine and blood urea nitrogen measurement

Automated chemistry analyzer for the assessment of serum creatinine (Scr) and blood urea nitrogen (BUN) levels (AU480, Beckman Coulter, Pasadena, CA). The Scr levels were presented as mg/dl, and the BUN levels were presented as mmol/L.

### Co-immunoprecipitation (Co-IP)

NRK-49F cells were lysed using 500 μl of NP40 lysis buffer on ice for 30 min and were then harvested using scrapper, followed by centrifugation. The supernatant containing equal amount of protein was incubated with primary antibodies for 1 h. Thereafter, the protein A/G PLUS-Agarose beads (sc-2003; Santa Cruz Biotech.) were added into the supernatant and incubated at 4 °C overnight. The immune precipitates were washed three times and subjected to Western blotting analysis.

### Cell co-culture

Raw264.7 cells were seeded into the upper chambers of a 24-well transwell plate and stimulated by TGF-β1 at 2 ng/ml. Six hours later, the supernatant was removed and replaced with a fresh medium without TGF-β1. NRK-49F cells were then seeded into the lower chamber in the absence or presence of Saracatinib. We then placed the upper chambers and lower chambers together. After 48 h, NRK-49F cell lysates were prepared and analyzed by Western blot.

### Statistical analysis

Statistical analysis of the data in this study was performed using IBM SPSS statistical software. All quantitative data were expressed as mean ± SEM. Comparisons between two groups were performed using two-tailed Student's t-test. Comparisons between three or more groups were analyzed by one-way ANOVA. Correlations between urinary Vtn and other index of kidney function were assessed using nonparametric Spearman correlation tests. A value of *P* < 0.05 was considered significant.

## Results

### Vtn is an integral component of the ECM scaffold in fibrotic kidney

**Figure 1:**
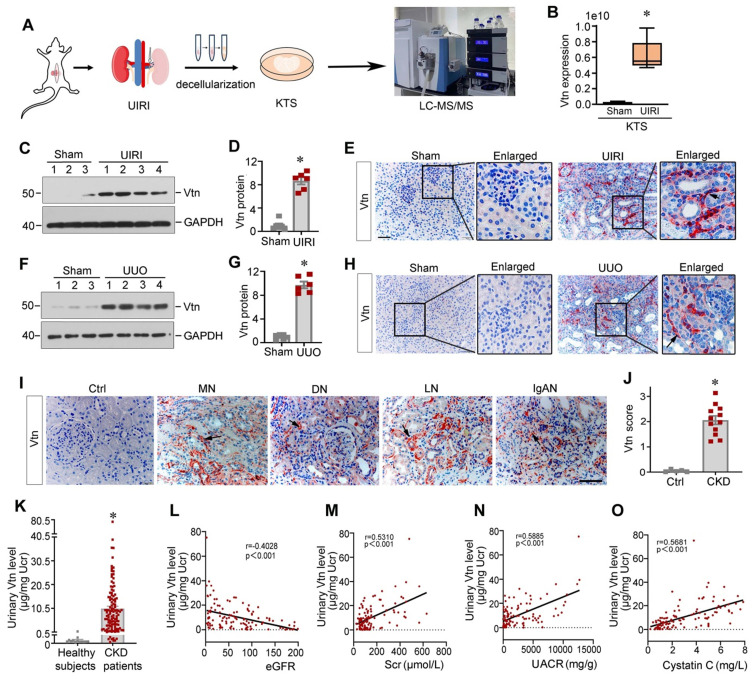
** Vitronectin is induced in chronic kidney disease in mice and human.** (A) The schematic presentation shows the discovery of vitronectin (Vtn) as one of the most upregulated proteins in the KTS of mice with unilateral ischemia-reperfusion injury (UIRI). Identification of protein expression in KTS samples was analyzed by liquid chromatography tandem mass spectrometry (LC-MS/MS). (B) Relative expression level of Vtn in the KTS of Sham and UIRI groups. ^*^P < 0.05 vs. sham group (n = 3). (C, D) Vtn expression in UIRI kidneys as detected by Western blot (C). Densitometric quantification (D) of renal Vtn expression in UIRI mice (n = 6). (E) Representative Vtn immunohistochemical staining in the kidney section from Sham and UIRI group. Arrow indicates positive Vtn expression. (F, G) Vtn expression in UUO kidneys as detected by Western blot (F). Densitometric quantification (G) of Vtn protein in the obstructed kidneys. (H) Immunohistochemical staining shows the expression of Vtn in Sham and UUO mice. The black arrow indicates Vtn-positive area. (I) Representative Vtn immunohistochemical staining in normal kidney and renal biopsies of various human CKD. Ctrl, the paraffin-tissues were derived from the non-tumorous kidney tissues of patients with renal cell carcinoma. Specimens from CKD patients with various etiologies, such as membranous nephritis (MN), diabetic nephropathy (DN), lupus nephritis (LN), and IgA nephropathy (IgAN). The black arrowheads indicate Vtn-positive staining. (J) Quantification of kidney Vtn expression in human biopsy tissues from healthy subjects and CKD patients. (K) Vtn was detected in urine in healthy subjects (n = 13) and CKD patients with various etiologies (n = 116). Urinary Vtn protein concentrations were corrected by urinary creatinine (µg/mg creatinine). (L-O The) Linear regression revealed that urinary Vtn were correlated with kidney dysfunction (estimated glomerular filtration rate [eGFR]) (L), serum creatinine (Scr) (M), urinary albumin creatinine ratio (UACR) (N) and serum cystatin C (O). ^*^P < 0.05 vs. sham controls or healthy subjects (n = 6-12). Scale bar, 50 µm.

To investigate the expression profile of the ECM proteins in CKD, we prepared decellularized KTS from normal and fibrotic mice after UIRI and carried out an unbiased quantitative proteomics analysis (Figure 1A) 19. By detecting the differential expression of ECM proteins, we identified that Vtn was one of the most upregulated proteins in the KTS of UIRI mice, compared to normal controls (Figure 1B).

To validate these proteomic data in vivo, we established two classic models of CKD induced by UIRI and UUO, respectively. Vtn expression was markedly increased after UIRI and UUO (Figure 1C-H). Immunohistochemical staining revealed that Vtn was unevenly distributed in renal interstitial compartment, but not in tubular epithelium (Figure 1E and H). These findings illustrate that Vtn is focally induced in distinct locations across kidney parenchyma after either ischemic or obstructive injury.

### Vtn is induced in human CKD and urinary Vtn correlates with kidney dysfunction

To establish the clinical relevance of Vtn induction to humans, we then examined Vtn expression in the kidneys of CKD patients by immunohistochemical staining. In non-tumorous adjacent kidney sections from renal cell carcinoma patients, Vtn protein staining was barely observable. However, strong Vtn expression was detected in the kidney biopsies from patients with various CKDs, including membranous nephritis (MN), diabetic nephropathy (DN), lupus nephritis (LN) and IgA nephropathy (IgAN). Vtn was primarily localized in renal interstitial area in CKD patients (Figure 1I-J), consistent with the findings from mouse models of CKD.

As Vtn is a secreted protein, we wondered whether it is present in the urine. We measured urinary Vtn levels in 13 healthy subjects and a cohort of 116 CKD patients by an enzyme-linked immunosorbent assay (ELISA). As shown in Figure 1K, the urinary level of Vtn protein markedly increased in CKD patients with different etiologies, compared to healthy subjects. Furthermore, we observed an inverse correlation between estimated glomerular filtration rate (eGFR) and urinary Vtn levels in 116 CKD patients (Figure 1L). Similarly, urinary Vtn protein was closely correlated with serum creatinine level (Figure 1M), urinary albumin creatinine ratio (Figure 1N), and cystatin C (Figure 1O). These results indicate that urinary Vtn level may serve as a potential non-invasive biomarker for kidney injury and dysfunction.

### Macrophage-derived Vtn promotes fibroblast activation and proliferation

**Figure 2:**
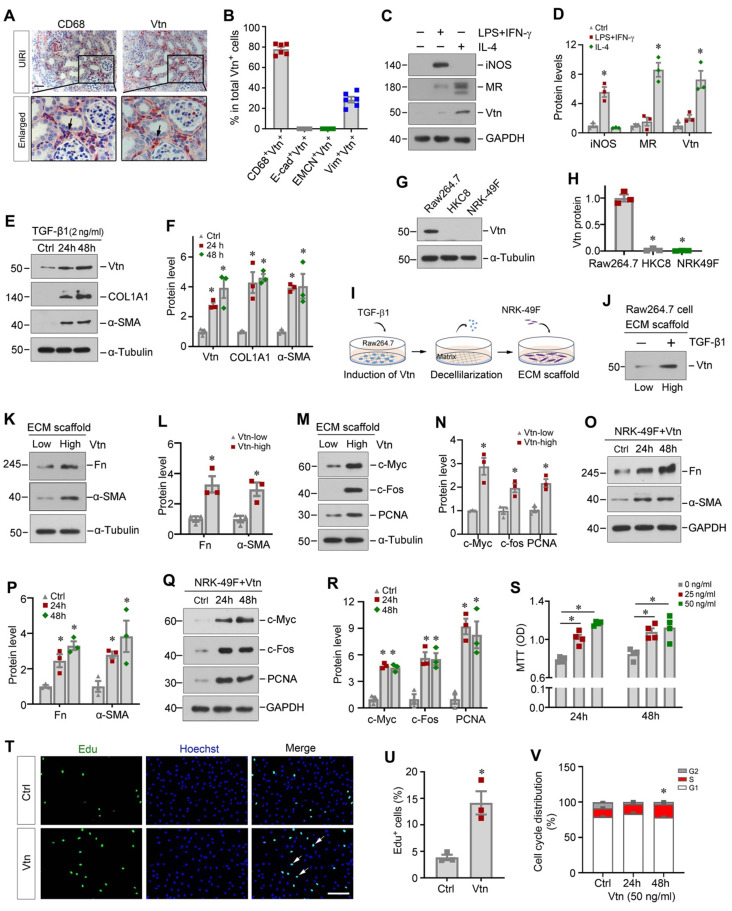
** Macrophage-derived Vtn promotes fibroblast activation in vitro.** (A) Immunohistochemical staining on adjacent serial sections showed co-localization of Vtn and CD68. Arrowheads indicate Vtn and CD68 positive cells. (B) Quantification of the percentage of the cells with double-staining for different cell markers (CD68, E-cadherin, EMCN, and vimentin) and Vtn. (C) Western blotting showed that the protein levels of iNOS (marker of M1 macrophages), mannose receptor (MR, markers of M2 macrophages) and Vtn after LPS plus IFN-γ or IL4 treatment. (D) Densitometric quantification of iNOS, MR and Vtn. ^*^P < 0.05 vs. control cells (n = 3). (E) Protein expression of Vtn, α-smooth muscle actin (α-SMA) and collagen I (COL1A1) in TGF-β1-treated Raw264.7 cells for 24 and 48 h. (F) Densitometric quantification of Vtn, COL1A1 and α-SMA. ^*^P < 0.05 vs. control cells (n = 3). (G) Western blot analysis showed that TGF-β1 induced Vtn only in Raw264.7, but not in NRK-49F and HKC8 cells. Various cells as indicated were treated with TGF-β1 at 2 ng/ml for 48 h. (H) Densitometric quantification of Vtn in various cells. ^*^P < 0.05 vs. Raw264.7 cells (n = 3). (I) Schematic presentation shows the experimental design for investigating the effects of macrophage-derived extracellular matrix (ECM) scaffold on renal fibroblasts. (J) Western blot showed the presence of Vtn protein in Raw264.7-derived ECM scaffold after TGF-β1 treatment. (K, L) Protein expression bands and densitometric quantification showed fibronectin (Fn) and α-SMA expression increased in NRK-49F cells seeded on Raw264.7-derived ECM scaffolds. Vtn-low, ECM scaffold prepared from control Raw264.7 cells; Vtn-high, ECM scaffold prepared from TGF-β1-treated Raw264.7 cells. (M, N) Western blots analysis showed an increased cell proliferation-related proteins in fibroblasts seeded on Raw264.7-derived ECM scaffolds. (O, P) Protein expression bands and densitometric quantification show that human recombinant Vtn induced α-SMA and Fn expression in NRK-49F cells. (Q) Human recombinant Vtn induced the expression of cell proliferation-related proteins in NRK-49F cells. (R) Densitometric quantification of c-Myc, c-fos and PCNA proteins. (S) Human recombinant Vtn at different concentrations (25 and 50 ng/ml) induced NRK-49F cell proliferation as detected by MTT assay. (T) EdU assay showed the effect of Vtn on the proliferation of NRK-49F cells. Scale bar, 100 µm. (U) Quantitative data of EdU-positive NRK-49F cells. ^*^P < 0.05 vs. control cells (n = 3). (V) Flow cytometry analysis showed that Vtn promoted cell cycle progression in NRK-49F cells. ^*^P < 0.05 vs. control cells (n = 3).

To identify the cellular origin of Vtn, we performed immunohistochemistry staining on adjacent serial sections. As shown in Figure 2A and B, Vtn was mainly co-localized with CD68, the macrophage marker, in the kidney of UIRI mice, indicating that Vtn was predominantly expressed in macrophages. A small fraction of Vtn^+^ cells was also stained positively with vimentin (Figure 2B). However, Vtn did not co-exist with epithelial marker E-cadherin or endothelial marker endomucin (EMCN) (Figure 2B, Figure S1A). To elucidate which subtype of macrophages responsible for producing Vtn, we induced macrophage polarization by incubating mouse monocyte/macrophage cell line (Raw264.7) with lipopolysaccharide and interferon-γ (LPS/IFN-γ) or interleukin-4 (IL-4), respectively. As shown in Figure 2C-D, Vtn was primarily produced by the mannose receptor (MR)^+^ M2 macrophages, while its induction in the inducible nitric oxide synthase (iNOS)^+^ M1 macrophages was marginal.

We found that Vtn was also induced in Raw264.7 cells in response to TGF-β1 stimulation (Figure 2E-F). Notably, TGF-β1 also induced the expression of collagen I (COL1A1) and α-smooth muscle actin (α-SMA) (Figure 2E-F). However, Vtn was not expressed in human proximal tubular epithelial cells (HKC-8) and kidney interstitial fibroblast cells (NRK-49F), even in the presence of TGF-β1 (Figure 2G-H), suggesting that Vtn is primarily produced in activated M2 macrophages in fibrotic kidney.

Given that Vtn is an ECM glycoprotein found in the KTS of fibrotic kidney (Figure 1A-B), we wondered whether Vtn contributes to the formation of the fibrogenic niche, leading to activation of the matrix-producing myofibroblasts. To test our hypothesis, we incubated Raw264.7 cells with TGF-β1 for 48 h to induce Vtn expression and secretion, and then prepared the ECM scaffold through decellularization (Figure 2I). NRK-49F cells were then seeded on Raw264.7-derived ECM scaffold and incubated for another 48 h. As shown in Figure 2J, Vtn protein was increased in the ECM scaffold from Raw264.7 cells treated with TGF-β1, compared to that from control group (Figure 2J). Of note, the ECM scaffold after decellularization protocol did not retain TGF-β1 protein (Figure S1B). We found that when fibroblasts cultivated on the Vtn-high ECM scaffold, they expressed an increased amount of fibronectin (Fn), α-SMA, c-Myc, c-Fos and proliferating cell nuclear antigen (PCNA) proteins (Figure 2K-N), indicating an enhanced activation of NRK-49F cells. These results suggest that Vtn contributes to the formation of the fibrogenic microenvironment that facilitates fibroblast activation and proliferation.

To directly confirm the role of Vtn in promoting fibroblast activation, we examined the effect of exogenous Vtn in regulating fibroblast activation using an in vitro system. Human recombinant Vtn directly induced Fn and α-SMA protein levels in fibroblasts (Figure 2O-P). Meanwhile, numerous proliferation-related proteins were induced by Vtn (Figure 2Q-R). Similarly, MTT assay showed that Vtn promoted NRK-49F cell proliferation in a time- and dose-dependent manner (Figure 2S). As shown in Figure 2T-U, Vtn also increased the incorporation of EdU, a nucleoside analog of thymine that is incorporated into DNA during active DNA synthesis 32, in NRK-49F cells after incubation for 48 h. Furthermore, we evaluated the potentiality of Vtn to promote cell cycle progression using flow cytometry analysis. Figure 2V illustrates the percentage of S phase increased in fibroblasts after incubation with Vtn, compared to the control. In summary, these data indicate that macrophage-derived Vtn may play an important role in mediating fibroblast activation.

### Knockout of Vtn ameliorates kidney fibrosis in vivo

**Figure 3:**
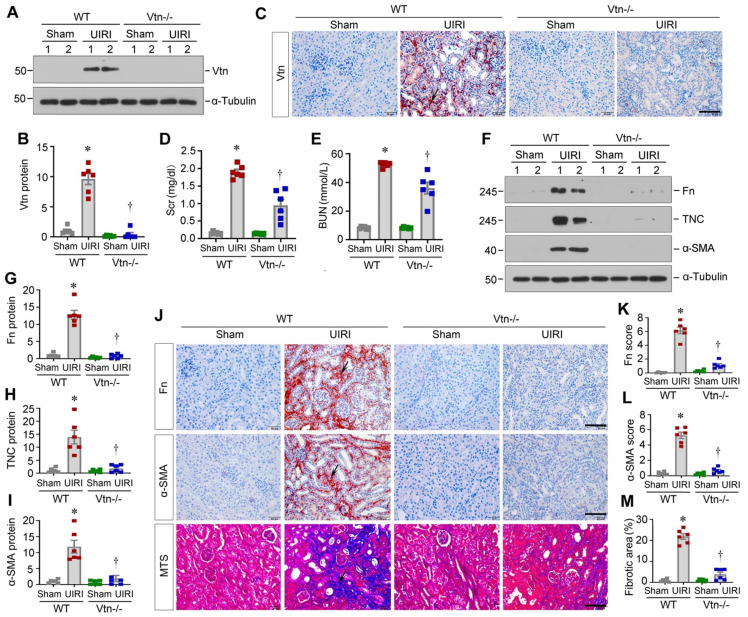
** Knockout of Vtn reduces renal fibrosis in mice after unilateral ischemia/reperfusion injury.** (A) Western blot analysis confirmed the ablation of Vtn in knockout mice. (B) Densitometric quantification of Vtn expression. (C) Immunohistochemical detection of Vtn in WT and Vtn KO mice. Arrow points to Vtn-positive area. (D, E) Serum levels of creatinine (Scr) and blood urea nitrogen (BUN) in WT and Vtn KO mice. (F-I) Western blot analysis showed the expression of fibrosis-related proteins (F). Densitometric quantification demonstrated renal expression of fibrosis-related proteins (G-I). (J) Immunohistochemical staining for Fn and α-SMA, and Masson's trichrome staining (MTS) for collagen deposition in WT and Vtn KO mice. Arrow points to positive area. (K-M) Quantitative determination of Fn, α-SMA and fibrotic lesions. ^*^P < 0.05 vs WT Sham group, ^†^*P* < 0.05 vs WT UIRI group (n = 6). Scale bar, 50 µm.

To validate whether Vtn is involved in the pathogenic process in renal fibrosis, we employed genetic Vtn knockout (Vtn-/-) mice. As displayed in Figure 3A-B, Vtn expression was evidently increased in wild-type (WT) mice after UIRI, but not in Vtn-/- mice. Immunohistochemical staining for Vtn produced the same result (Figure 3C). We found that serum creatinine (Scr) and blood urea nitrogen (BUN) levels were elevated in WT mice after UIRI. However, ablation of Vtn decreased Scr and BUN levels in Vtn-/- mice (Figure 3D-E).

We also examined renal fibrosis by analyzing the protein level of fibrosis-related proteins. Fn, TNC and α-SMA were induced in WT mice after UIRI, but not in Vtn-/- mice, suggesting that ablation of Vtn inhibits the expression of these proteins (Fig 3F-I). Similar results were observed in immunohistochemical staining for Fn and α-SMA (Figure 3J-L). Masson's trichrome staining (MTS) also exhibited a clear reduction in collagens deposition in the kidney of Vtn-/- mice, compared to WT controls (Figure 3J and M).

### Knockdown of Vtn attenuates kidney fibrosis after ischemic injury

**Figure 4:**
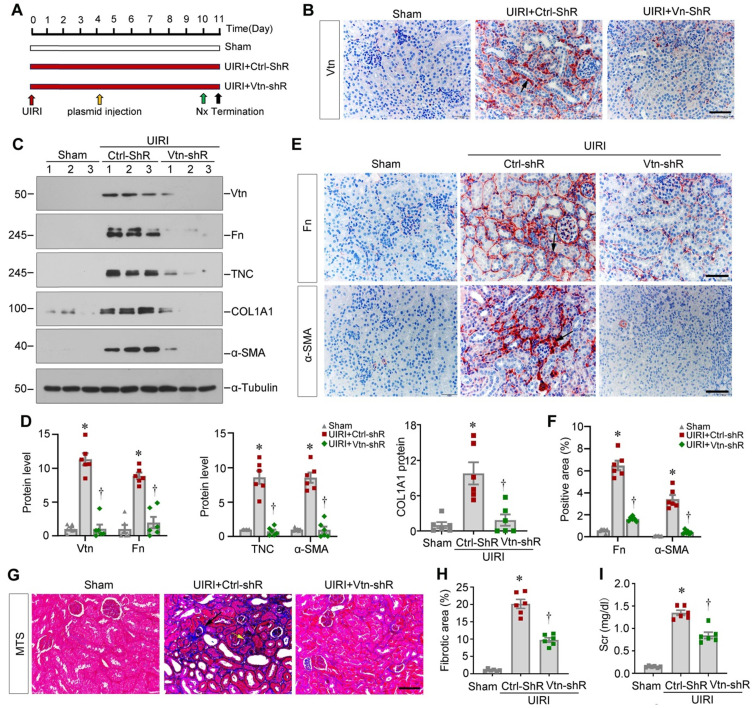
** Knockdown of Vtn alleviates kidney fibrosis after UIRI.** (A) Schematic illustration of experiment procedure. Red, green, and black arrowheads show the points of time for UIRI, nephrectomy (UNx), and sacrifice, respectively. The yellow arrow indicates injections of plasmid vectors carrying Vtn short hairpin RNA (shRNA) (pLVX-Vtn-shRNA) or negative control (Ctrl-shRNA) on day 4 after UIRI. (B) Representative immunohistochemical staining for Vtn. Arrow points to Vtn-positive area. (C) Representative protein expression bands show renal Vtn protein and fibrosis-related proteins (Fn, TNC, COL1A1, and α-SMA) expression in different groups. (D) Graphic presentations depict the relative abundance of renal Vtn, Fn, TNC, COL1A1 and α-SMA. (E, F) Representative micrographs (E) and quantification of positive area (F) showed Fn and α-SMA protein expression. Arrow points to Fn and α-SMA positive area. Scale bar, 50 µm. (G) Interstitial fibrotic lesion was assessed by MTS. Arrowheads points to collagen deposition area. (H) Quantitative determination of fibrotic area is presented. (I) Histogram shows Scr levels. ^*^P < 0.05 vs Sham group, ^†^*P* < 0.05 vs UIRI plus Ctrl-shR group (n = 6). Scale bar, 50 µm.

Apart from the Vtn-/- knockout genetic model, we next explored the effect of Vtn in kidney fibrosis through knocking down Vtn expression. To this end, mice were injected with Vtn-specific short hairpin RNA (Vtn-shRNA) plasmid or control vector (Ctrl-shRNA) via the tail vein, as previously reported 19, 30. Figure 4A shows the experimental design. Figure 4B demonstrates that injection of Vtn-shRNA decreased Vtn protein expression in the kidney of UIRI mice. Knockdown of Vtn clearly reduced Fn, TNC, COL1A1 and α-SMA protein levels in UIRI mice (Figure 4C-D). Immunohistochemical staining for Fn and α-SMA showed similar results (Figure 4E-F). MTS also demonstrates that knockdown of Vtn mitigated collagen deposition and fibrotic lesions in UIRI mice (Figure 4G-H). Consistently, the Scr level was reduced in UIRI mice after injection of Vtn-shRNA, compared to control vector (Ctrl-shR) (Figure 4I).

### Overexpression of Vtn aggravates kidney fibrosis in vivo

**Figure 5:**
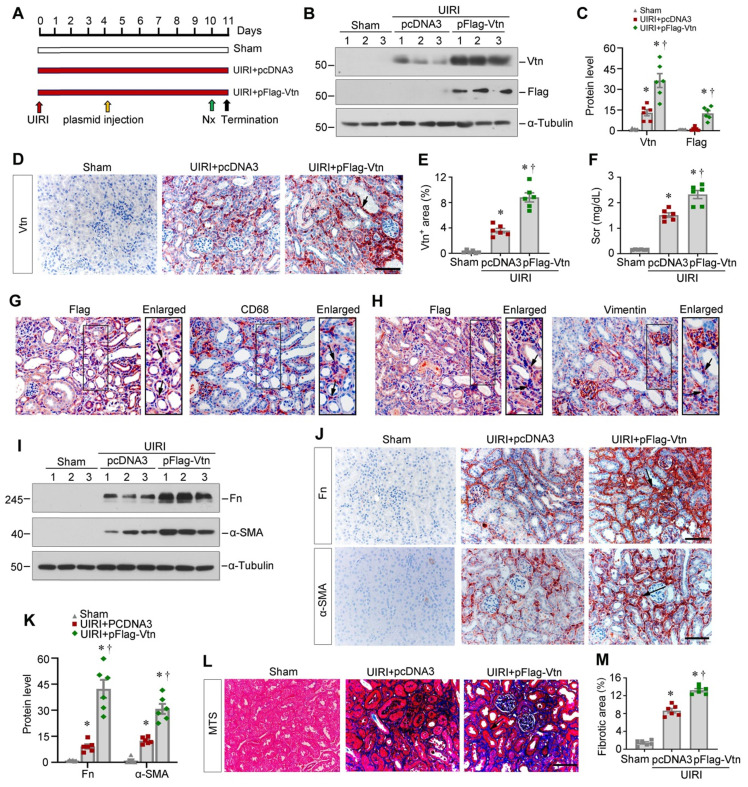
** Exogenous Vtn aggravates kidney fibrosis after UIRI.** (A) Schematic illustration of experimental protocol. Red, green, and black arrowheads show the points of time for UIRI, UNx and sacrifice, respectively. The yellow arrow indicates the injection of empty vectors (pcDNA3) or Flag-tagged Vtn expression plasmid (pFlag-Vtn) on day 4 after UIRI. (B) Western blot results of renal Vtn and Flag. (C) Densitometric quantification of renal Vtn and Flag expression in different groups as indicated. (D, E) The expression of Vtn in different groups. Arrowhead points to Vtn-positive area. (F) Graph shows Scr levels in different groups as indicated. (G) Immunohistochemical staining for Flag and CD68 was performed on adjacent serial sections. Arrow indicates colocalization of Flag and CD68. (H) Immunohistochemical staining for Flag and vimentin was performed on adjacent serial sections. Arrow indicates colocalization of Flag and vimentin. (I, K) Representative protein expression bands (I) and densitometric quantification (K) show Fn and α-SMA expression. (J) Representative images show Fn and α-SMA in each group. Arrows indicate Fn and α-SMA positive area. (L) Representative MTS pictures of kidney fibrosis in different groups. Arrowhead points to collagen deposition area. (M) Histogram shows the quantitative determination of collagen deposition area in different groups. ^*^P < 0.05 vs sham operation group; ^†^*P* < 0.05 vs UIRI plus pcDNA3 group (n = 6). Scale bar, 50 µm.

To directly confirm the effect of Vtn, we further sought to overexpress exogenous Vtn. We delivered Flag-tagged Vtn expression vector (pFlag-Vtn) via tail vein injection. Figure 5A shows the experimental design. The efficiency of Vtn overexpression in vivo was validated by Western blot analysis of Vtn and Flag, respectively (Figure 5B-C). Figure 5D and E show that Vtn was upregulated mainly in renal interstitium at 11 days after UIRI, compared with pcDNA3-injected group. This increase in Vtn expression was associated with an elevated level of Scr in UIRI mice, suggesting that overexpression of Vtn aggravates kidney dysfunction (Figure 5F). Immunohistochemical staining for Flag and CD68 on serial sections demonstrated their co-localization (Figure 5G), indicating that exogenous Flag-tagged Vtn transgene was targeted to macrophages by this approach. Similarly, Flag-Vtn fusion protein was also detected in some vimentin-positive fibroblast cells (Figure 5H).

Overexpression of exogenous Vtn elevated the protein levels of Fn and α-SMA in the kidneys after UIRI (Figure 5I and K). Immunohistochemical staining also confirmed that overexpression of Vtn aggravated Fn and α-SMA expression in fibrotic kidney (Figure 5J). MTS presented an increased collagen deposition in UIRI mice injected with pFlag-Vtn, compared to pcDNA3 group (Figure 5L-M).

### Vtn induces fibroblast activation via stimulating integrin αvβ5/Src signaling

**Figure 6:**
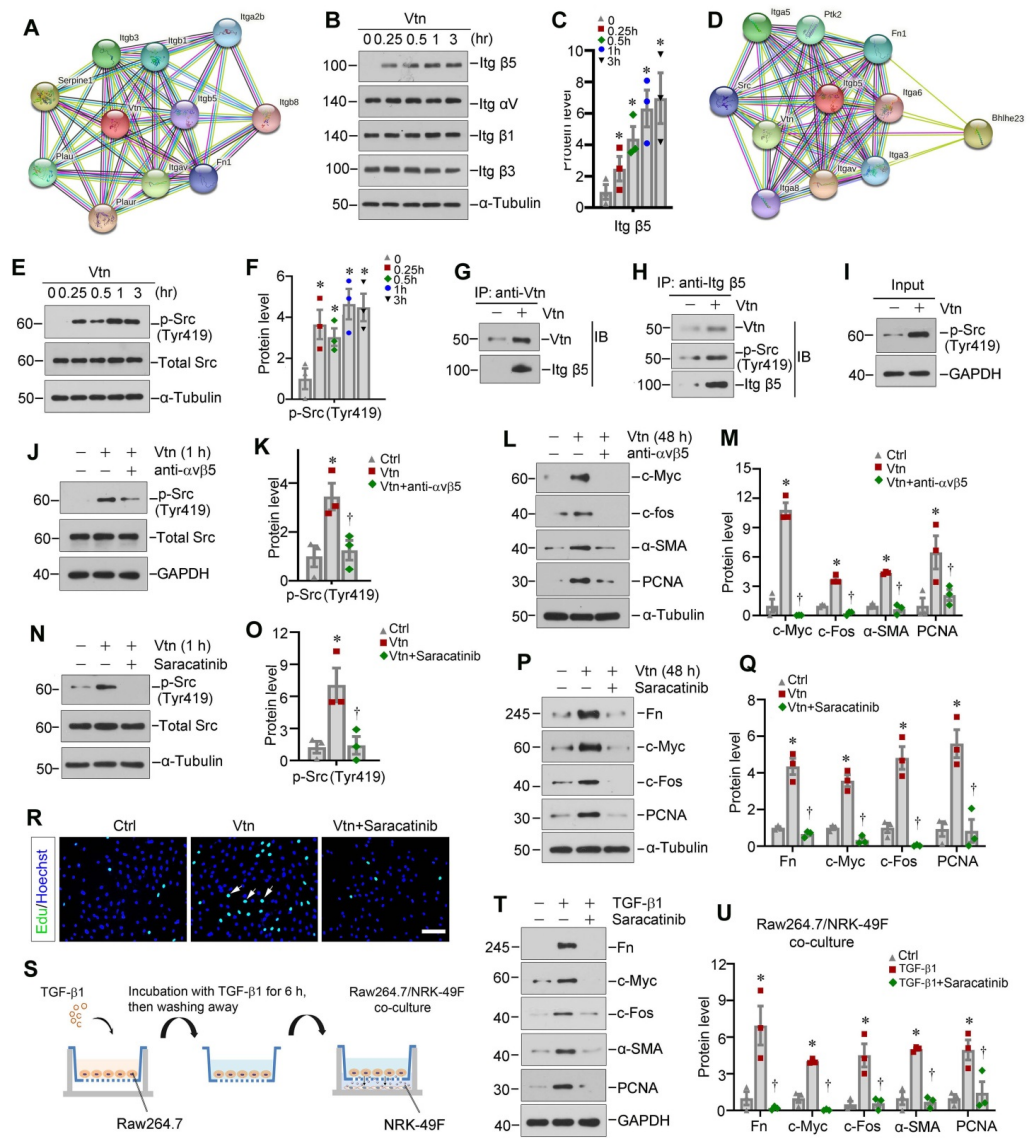
** Vtn activates integrin αvβ5/Src signaling in vitro.** (A) The protein-protein interaction (PPI) network of Vtn revealed by STRING analysis. (B) Representative Western blots show integrin αv, β1, β3 and β5 expression in human Vtn (50 ng/ml)-stimulated NRK-49F cells at different time points. (C) Quantitative data of protein bands show integrin β5 (Itg β5) expression in Vtn-stimulated NRK-49F cells. ^*^P < 0.05 vs control cells (n = 3). (D) The PPI networks of integrin β5 revealed by STRING analysis. (E, F) Representative images of protein bands (E) and densitometric quantification (F) show p-Src (Tyr419) and total Src expression. ^*^P < 0.05 vs. control cells (n = 3). (G-I) Co-immunoprecipitation demonstrates that Vtn formed a complex with integrin β5 and p-Src (Tyr419) in NRK-49F cells. (J, K) Representative protein expression bands (J) and densitometric quantification (K) show p-Src (Tyr419) and total Src expression in Vtn-stimulated NRK-49F cells in the absence or presence of integrin αvβ5 blocking antibody for 1 h. ^*^P < 0.05 vs normal NRK-49F cells, ^†^*P* < 0.05 vs Vtn alone (n = 3). (L, M) Representative protein expression bands (L) and densitometric quantification (M) show an increased expression of proliferation-related proteins. ^*^P < 0.05 vs normal NRK-49F cells, ^†^*P* < 0.05 vs Vtn alone (n = 3). (N, O) Western blots (N) and quantification (O) show p-Src (Tyr419) and total Src expression in Vtn-stimulated NRK-49F cells with or without of Src inhibitor Saracatinib for 1 h. ^*^P < 0.05 vs normal NRK-49F cells, ^†^*P* < 0.05 vs Vtn alone (n = 3). (P, Q) Western blots (P) and densitometric quantification (Q) show the proliferation-related proteins. ^*^P < 0.05 vs normal NRK-49F cells, ^†^*P* < 0.05 vs Vtn alone (n = 3). (R) Representative images of EdU-positive NRK-49F cells. (S) Diagram shows the Raw264.7/NRK49F cells co-culture experiment. Raw264.7 cells were induced to secrete Vtn by priming with TGF-β1. After 6 h of incubation, TGF-β1 was removed by changing to fresh media. NRK-49F cells were co-cultured with Raw264.7 cells in different chambers in the absence or presence of Saracatinib for 48 h. (T, U) Western blots (T) and densitometric quantification (U) show fibrosis-related and cell proliferation-related proteins expression in NRK-49F cells after co-culture with Raw264.7 cells. ^*^P < 0.05 vs normal NRK-49F cells, ^†^*P* < 0.05 vs NRK-49F cells co-culture with TGF-β1-treated Raw264.7 cells (n = 3). Scale bar, 100 µm.

To identify potential signaling pathway involved in mediating Vtn action, we first explored the interacting partners of Vtn by analyzing the protein-protein interaction (PPI) network using STRING program. Figure 6A shows the results of STRING analysis, which revealed the potential of Vtn to interact with various integrins. Based on this finding, we experimentally confirmed that treatment of NRK-49F cells with Vtn increased integrin β5, but not αv, β1 and β3 (Figure 6B-C, and Figure S2A). We then generated a PPI network of integrin β5 using STRING and found both Vtn and non-receptor tyrosine kinase Src as potential interacting partners of integrin β5 (Figure 6D).

Based on these data, we experimentally confirmed that Vtn caused a rapid phosphorylation of Src kinase on Tyr419 (Figure 6E-F). Furthermore, we performed co-immunoprecipitation (Co-IP) to determine Vtn/integrin β5/p-Src complex formation. As shown in Figure 6G-I, the complex formation of Vtn, integrin β5 and Src kinase was evident in NRK-49F cells, as Vtn, integrin β5 and p-Src could be found in the immunocomplexes precipitated by anti-integrin β5 antibody.

We further investigated the role of integrin αvβ5/Src signal cascade in mediating Vtn action. Incubation of NRK-49F cells with integrin αvβ5 blocking antibody largely inhibited the Vtn-induced upregulation of p-Src (Tyr419) (Figure 6J-K). Moreover, blockade of integrin αvβ5 abolished the expressions of c-Myc, c-Fos, α-SMA and PCNA induced by Vtn (Figure 6L-M).

To identify whether Src kinase activation in mediating Vtn action, we employed Saracatinib, a selective tyrosine kinase inhibitor of Src kinase 33, 34. As shown in Figure 6N-O, pretreatment with Saracatinib blocked Src phosphorylation at Tyr419 in NRK-49F cells. Furthermore, Saracatinib also abrogated Vtn-induced Fn, c-Myc, c-Fos and PCNA expression (Figure 6P-Q). MTT assay, EdU incorporation assay and cell cycle analysis showed that inhibition of Src signaling by Saracatinib repressed NRK-49F cell proliferation in response to Vtn (Figure 6R and Figure S2B-D).

To closely mimic the in vivo environment of fibrotic kidney, we co-cultured Raw264.7 macrophages with NRK-49F fibroblast cells. The experimental design is illustrated in Figure 6S. NRK-49F cells activation and proliferation were induced by TGF-β1-primed macrophage after co-culture, which was suppressed by Saracatinib (Figure 6T-U). Similarly, co-culture with TGF-β1-primed macrophages induced NRK-49F cell proliferation as assessed by EdU incorporation, which was also inhibited by Saracatinib (Figure S2E-F).

### Vtn promotes interstitial fibroblast proliferation via integrin αvβ5/Src signaling in vivo

**Figure 7:**
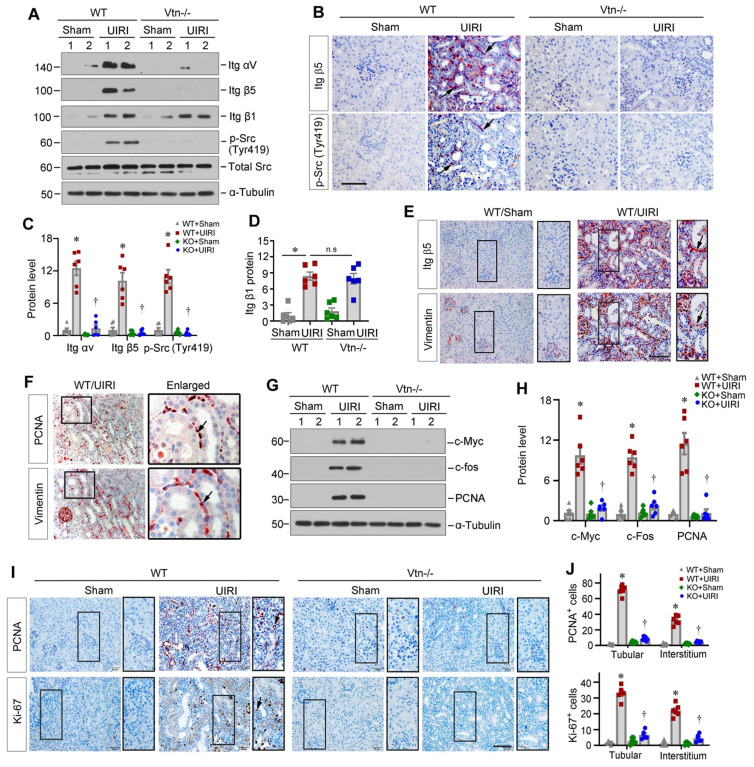
** Knockout of Vtn inhibits integrin αvβ5/Src signaling in UIRI model.** (A) Western blots show integrin αv, integrin β5, integrin β1, p-Src (Tyr419) and total Src expression in WT and Vtn KO mice. (B) Immunohistochemical staining on adjacent serial sections showed co-localization of integrin β5 and p-Src (Tyr419). Arrowheads point to positive staining. Scale bar, 50 µm. (C, D) Histograms show integrin αv, integrin β5, p-Src (Tyr419) (C) and integrin β1 (D) expression in WT and Vtn KO mice. (E) Immunohistochemical staining for integrin β5 and vimentin on adjacent serial sections. Arrowheads point to co-localization. (F) Immunohistochemical staining for PCNA and vimentin on adjacent serial sections. (G, H) Protein expression bands (G) and densitometric quantification (H) show proliferation-related proteins expression. (I, J) Immunohistochemical staining (I) and the positive cell numbers (J) of PCNA and Ki-67 in each group. ^*^P < 0.05 vs WT Sham group, ^†^*P* < 0.05 vs WT UIRI group, n.s, not significant (n = 6). Scale bar, 50 µm.

We further assessed whether Vtn activates the integrin αvβ5/Src signaling in kidney fibroblasts in vivo. Protein levels of renal integrin αvβ5 and p-Src (Tyr419) were increased in WT mice after UIRI, but not in Vtn-/- kidneys (Figure 7A-C). However, renal mRNA expression of integrin αv and β5 was not changed in different groups (Figure S3A), suggesting that Vtn upregulates integrin αvβ5 primarily by a posttranscriptional regulation. The expression level of integrin β1 was also increased after UIRI in both WT and Vtn-/- mice (Figure 7A and D), suggesting that activation of avβ5 and Src, but not β1, was dependent on Vtn in the fibrotic kidney. Immunohistochemical staining for integrin β5 and p-Src demonstrated their co-localization in the interstitium of the kidneys after UIRI in WT but not Vtn-/- mice (Figure 7B), suggesting that this pathway is activated in the same interstitial cells. We also observed similar downregulation of integrin αvβ5/Src signaling in the Vtn knockdown model (Figure S3B-D). To further confirm the identity of these integrin β5^+^/p-Src^+^cells, we performed immunohistochemical staining on serial sections for integrin β5 and vimentin, a marker of activated fibroblasts. As shown in Figure 7E, integrin β5 largely co-localized with vimentin, indicating that fibroblast cells responded to Vtn, leading to integrin β5 activation and p-Src phosphorylation. We further studied the consequence of Src activation by examining fibroblast proliferation via immunohistochemical staining on adjacent serial sections for PCNA and vimentin. As shown in Figure 7F, PCNA^+^ cells were largely overlapping with vimentin^+^ fibroblasts in UIRI mice, suggesting active fibroblast proliferation.

We further examined the impact of Vtn on fibroblast activation by using Western blotting. IRI caused marked induction of renal proliferation-related proteins (Figure 7G-H). However, ablation of Vtn in Vtn-/- mice completely abolished their induction. Furthermore, immunohistochemical staining for PCNA and Ki-67 revealed marked induction of proliferating cells in both tubular and interstitial compartments of the kidneys in WT mice. However, ablation of Vtn abolished cell proliferation after UIRI (Figure 7I-J). Consistently, overexpression of exogenous Vtn in mice aggravated avβ5 and p-Src activation, c-Fos and PCNA induction and cell proliferation after UIRI (Figure S4).

### Pharmacologic blockade of Src inhibits fibroblast proliferation and attenuates renal fibrosis

**Figure 8:**
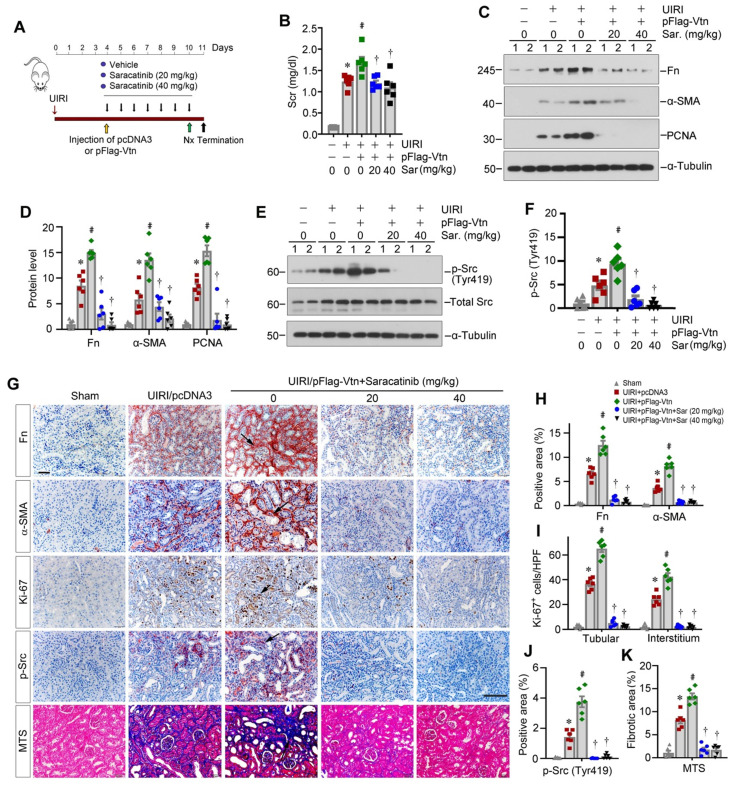
** Pharmacologic blockade of Src signaling inhibits fibroblast proliferation and attenuates renal fibrosis in vivo.** (A) A schematic diagram illustrating experimental protocol. Red, green, and black arrowheads show the points of time for UIRI, UNx and sacrifice, respectively. The yellow arrowhead denotes the mouse tail-vein injection of empty vector (pcDNA3) or Flag-tagged Vtn expression plasmid (pFlag-Vtn). Repeated black arrowheads indicate the points of time when mice were treated with different doses of Saracatinib by intraperitoneal injection. (B) Histogram displays Scr levels in five groups as indicated. (C, D) Western blots and densitometric quantification demonstrate Fn, α-SMA and PCNA expression. (E, F) Western blots (E) and densitometric analysis (F) show p-Src (Tyr419) and total Src expression in different groups. (G) Immunohistochemical staining for Fn, α-SMA, Ki-67, p-Src (Tyr419) and MTS for collagen deposition. Arrowheads represent positive area. (H-K) Graphs depict the percentage of Fn, α-SMA (H) and p-Src (Tyr419) (J) positive area and the total number of Ki-67^+^ (I) cells. Quantitative data of kidney fibrosis area (K) in each group. At least 10 fields of kidney sections were randomly selected, and the average of positive area per mouse was calculated. ^*^P < 0.05 vs sham group, ^#^*P* < 0.05 vs UIRI plus pcDNA3 group, ^†^*P* < 0.05 vs UIRI plus pFlag-Vtn group (n = 6). Scale bar, 50 µm.

To ascertain Src mediating Vtn-triggered renal fibrosis, we chose to use Src inhibitor Saracatinib. Mice were treated with Saracatinib through intraperitoneal injections at the doses of 20 and 40 mg/kg/day, respectively. Figure 8A shows the experimental protocol. After injection of pFlag-Vtn plasmid, Scr was increased in UIRI mice. However, inhibition of Src signaling by Saracatinib attenuated the detrimental effects of Vtn on kidney function (Figure 8B). Saracatinib also suppressed the expression of Fn, α-SMA and PCNA proteins in UIRI plus pFlag-Vtn group (Figure 8C-D). As speculated, Saracatinib inhibited the phosphorylation of Src kinase (Figure 8E-F). Consistently, immunohistochemical staining also revealed that Saracatinib inhibited protein levels of Fn, α-SMA, Ki-67 and p-Src in UIRI mice injected with pFlag-Vtn (Figure 8G-J). Finally, Saracatinib alleviated collagen deposition in UIRI mice injected with pFlag-Vtn, as shown by MTS (Figure 8G and K).

## Discussion

Kidney fibrosis is a step-wise process, in which the establishment of the fibrogenic niche is an initial step. This is followed by fibroblast expansion, and subsequent deposition and accumulation of ECM, resulting in generation of fibrotic foci throughout renal parenchyma 15, 16. However, the molecular composition of fibrogenic niche and its underlying mechanisms remain largely elusive. Using decellularized KTS coupled with proteomic profiling, we identified Vtn as a crucial component of the fibrogenic microenvironment. Several lines of evidence support this conclusion. i) Vtn is upregulated and secreted primarily by macrophages in various models of CKD. ii) Urinary Vtn levels increase in patients with CKD and inversely correlates with kidney function. iii) Extracellular Vtn in ECM scaffold promotes fibroblast activation and induces their proliferation. iv) Vtn deficiency or depletion hampers fibroblast activation and proliferation in vivo, while overexpression of Vtn aggravates fibroblast activation and kidney fibrosis. v) Vtn activates the integrin β5/Src kinase in renal fibroblasts in vitro and in vivo. vi) Blockade of integrin αvβ5 or pharmacologic inhibition of Src suppresses fibroblast activation and ameliorates kidney fibrotic lesions. Collectively, these studies illustrate that macrophage-derived Vtn can promote fibroblast activation and expansion by assembling a profibrotic microenvironment. Our findings suggest a previously unrecognized mechanism by which macrophages contribute to fibroblast activation and kidney fibrosis.

Macrophage infiltration is a central event in tissue fibrosis, which usually correlates with the severity of fibrotic lesions 10, 13. How macrophages contribute to fibroblast activation and expansion, however, is ambiguous and sometimes controversial. Some studies have shown that macrophages may directly transdifferentiate into α-SMA-positive, matrix-producing myofibroblasts by a process known as macrophage-myofibroblast transition (MMT) 35, 36. Other reports suggest that macrophages may regulate fibroblast activation in a paracrine manner by secreting soluble factors 8, 37. As macrophages and fibroblasts are localized nearby in the interstitial compartment, such a close proximity enables them to influence each other via soluble cues such as cytokines, chemokines and growth factors. Here we present a completely new scenario in which forming a particular extracellular microenvironment serves as a novel means for mediating macrophage-fibroblast interaction. In this circumstance, macrophages produce and secrete ECM glycoprotein Vtn, which becomes an integral composition of the fibrogenic niche. This in turn provides a permissive microenvironment for fibroblasts to proliferate and secrete a large amount of ECM proteins, leading to scar formation. Therefore, the present study advocates a previously unrecognized mode of cell-cell interaction in which macrophages promote fibroblast activation by secreting ECM protein and assembling a special microenvironment 9. It should be pointed out that the findings presented in this study by no means exclude the importance of other soluble factors released by macrophages in mediating fibroblast activation and kidney fibrosis.

Vtn is a glycoprotein present in serum and the ECM compartment and involves in numerous biological and pathological processes 20, 38. Vtn is absent in most normal tissues but increases in areas of fibrosis in various disorders 22, 26. Earlier studies have shown an increased Vtn expression in atherosclerotic lesions and acute myocardial infarction 20. An extensive accumulation of Vtn is also noticed in rheumatoid arthritis, lung fibrosis and UUO 26, 39. Furthermore, previous work has shown that urinary Vtn levels are elevated in a cohort of kidney transplant recipients and inversely correlate with kidney function 24. In this study, we found that Vtn is one of the most upregulated proteins in the KTS of fibrotic kidney, as uncovered by mass spectrometry (Figure 1). These data suggest that Vtn is an integral element of the fibrogenic niche that aggravates fibroblast activation. As a secreted protein, Vtn can be easily detected in urine samples of CKD patients with various kinds of etiologies, suggesting that it may serve as a non-invasive biomarker for estimating and monitoring the progression of renal fibrosis. Indeed, we show that urinary Vtn is increased in CKD patients and its levels closely corelate with the severity of kidney insufficiency (Figure 1), underscoring that urinary Vtn can serve as a non-invasive biomarker for CKD progression.

The present study demonstrates that Vtn is induced primarily in activated M2 macrophages, whereas it is undetectable in tubular cells and endothelial cells in the fibrotic kidneys (Figure 2 and S1). A small fraction of Vtn^+^ cells express vimentin, implying that Vim^+^ fibroblasts may also express Vtn, although in vitro Vtn is only detected in Raw264.7 cells but not in HKC-8 and fibroblasts, even after TGF-β1 stimulation (Figure 2G). At this stage, it remains unknown whether these Vtn^+^/Vim^+^ cells represent a subpopulation of Vtn-producing fibroblasts or Vtn-producing macrophages acquiring vimentin during their transition to myofibroblasts via MMT 35, 36. Renal fibroblasts reside in the interstitial compartment and play a vital role in ECM homeostasis 7. However, after kidney injury, the quiescent fibroblasts become activated, which is characterized by two predominant features: proliferation and myofibroblastic transformation 5, 40, 41. Using both loss- or gain-of-function approaches, this study offers unambiguous evidence for Vtn in mediating fibroblast activation and proliferation. In vivo, depletion of Vtn alleviates UIRI-triggered fibroblast activation and proliferation, which is accompanied by renal function recovery and inhibition of Fn, COL1A1, α-SMA, c-Myc, c-Fos, and PCNA. On the contrary, overexpression of Vtn accelerates fibroblast proliferation and kidney fibrosis (Figure 2-5 and 7). Of note, there was an inconsistent degree of protection between renal function and fibrosis-related markers in Vtn-/- mice (Figure 3). This is largely due to the fact that renal function parameters in this model may only reflect an impaired compensatory capacity of the injured kidney after UNx and it may not truly indicate renal failure 42. Nevertheless, a high level of Scr and BUN means an impaired compensatory capacity of the kidney following UNx. We demonstrate that recombinant Vtn or Vtn-high KTS induces fibroblast activation and expansion by in vitro and ex vivo approaches (Figure 2). Therefore, Vtn upregulation in macrophages after kidney injury elicits crucial effect on fibroblast activation and proliferation.

This study also delineates the mechanism by which Vtn promotes fibroblast activation and kidney fibrosis. Cell-matrix interactions control cell behaviors by triggering intracellular signal transduction through cell surface receptors. The integrins are a family of ECM receptors that transmit signals from microenvironment to the cell 43. Only three integrin complexes (integrins αvβ1, αvβ3 and αvβ5) are expressed on fibroblast cell surface and can be recognized by Vtn 20, 44. While integrins αv, β1, β3 and β5 are upregulated in the IRI mice, only the expression of integrin β5, but not αv, β1 and β3, is elevated in Vtn-treated NRK-49F cells (Figure 6), suggesting that integrin β5 is the principal receptor mediating Vtn action. Consistently, co-immunoprecipitation assay confirms that Vtn could bind to integrin β5. Of note, integrin β5 is never reported to pair with any other α integrin except the αv. In this context, it is reasonable to assume that Vtn exerts its pro-fibrotic action exclusively by binding to integrin αvβ5 (Figure 6 and 7).

The interaction of Vtn and integrin αvβ5 causes them to become clustered in cell membrane, leading to subsequent phosphorylation and activation of intracellular targets such as Src kinase. Src belongs to a family of nonreceptor protein tyrosine kinase and is required for the signaling triggered by integrins/ECM interaction 45. Several studies show that Src is a critical kinase for regulating fibroblast activation 11, 46, 47. These are in line with our present study showing that suppression of Src by Saracatinib, a small molecule Src kinase inhibitor 33, 34, abolished fibroblast activation and proliferation (Figure 6**)**. Furthermore, integrin β5 physically interacts with Vtn and Src as shown by co-immunoprecipitation (Figure 7), suggesting that Vtn/integrin β5 engagement triggers a large protein complex formation, leading to Src phosphorylation. Accordingly, inhibition of Src by Saracatinib dose-dependently mitigates kidney fibrosis in vivo (Figure 8). These findings support the notion that Vtn/integrin β5/Src signal cascade links macrophage to fibroblast activation in fibrotic kidney.

In summary, this study demonstrates that infiltrated macrophages after kidney injury secrete Vtn to extracellular space and assemble a profibrotic microenvironment, leading to fibroblast activation and expansion. These findings provide novel insights into macrophage/fibroblast interaction via Vtn/integrin β5/Src signaling. Our results also suggest that urinary Vtn can be a potential non-invasive biomarker for kidney fibrosis and targeting Vtn/integrin β5/Src signaling could pave a new avenue for treating fibrotic CKD.

## Supplementary Material

Supplementary figures and tables.

## Abbreviations

CKD: chronic kidney disease
COL1A1: collagen I
KTS: kidney tissue scaffold
ECM: extracellular matrix
MTS: masson's trichrome staining
α-SMA: α-smooth muscle actin
TNC: tenascin C
UIRI: unilateral ischemia-reperfusion injury
UUO: unilateral ureteral obstruction
Vtn: Vitronectin
