# Supplementary material for: Macrophage promotes fibroblast activation and kidney fibrosis by assembling a vitronectin-enriched microenvironment
Source: Theranostics. 2023 Jul 3;13(11):3897–913. doi: 10.7150/thno.85250 (PMC10334827; doi:10.7150/thno.85250)
Supplement: Supplementary file 1 — Supplementary figures and tables. [file thnov13p3897s1.pdf]

Macrophage promotes fibroblast activation and kidney fibrosis by assembling a vitronectin-enriched microenvironment

Yiling Peng*, Li Li*, Jingyue Shang, Haili Zhu, Jinlin Liao, Xue Hong, Fan Fan Hou, Haiyan Fu† and Youhua Liu†

Supplementary material

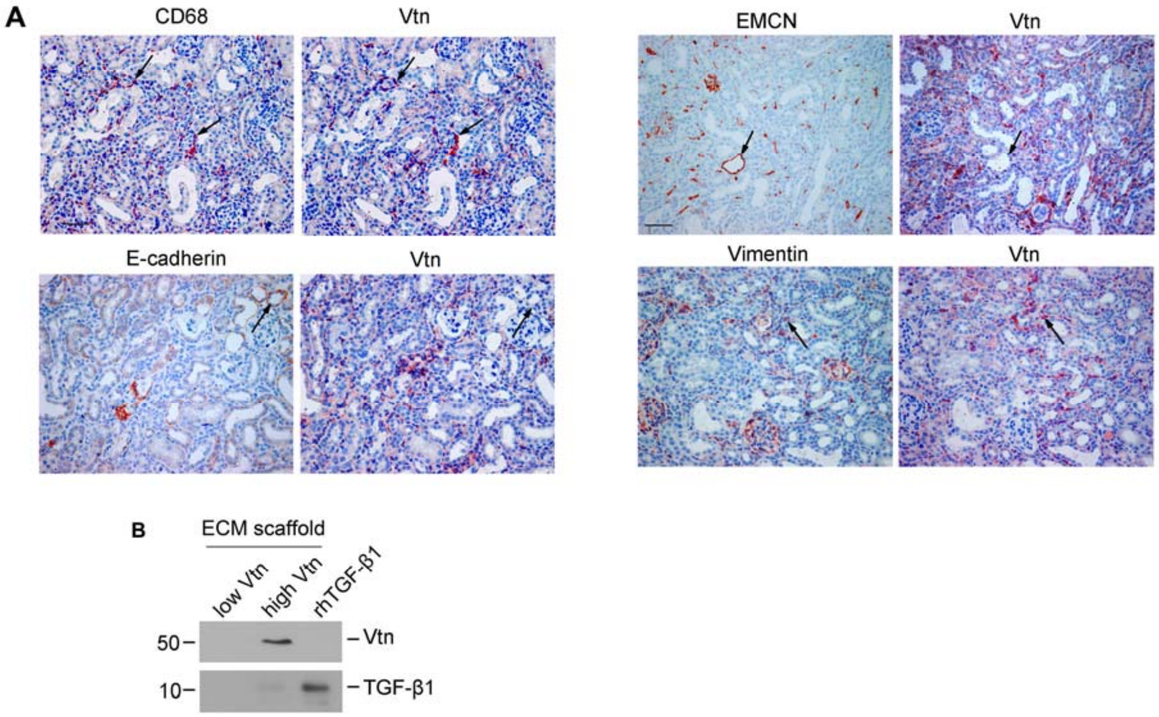

Supplementary Figure S1. The cellular source of Vtn. (A) Adjacent serial sections of the kidneys were stained with antibodies against Vtn and CD68 (macrophage marker), endomucin (EMCN, endothelial marker), E-cadherin (tubular cells marker), and vimentin (fibroblast marker), respectively. Arrows indicate the same areas on the serial sections. Scale bar, 50 μm. (B) Western blot showed the presence of Vtn protein and the absence of TGF-β1 in Raw264.7-derived ECM scaffold after TGF-β1 treatment. rhTGF-β1, recombinant human TGF-β1 protein.

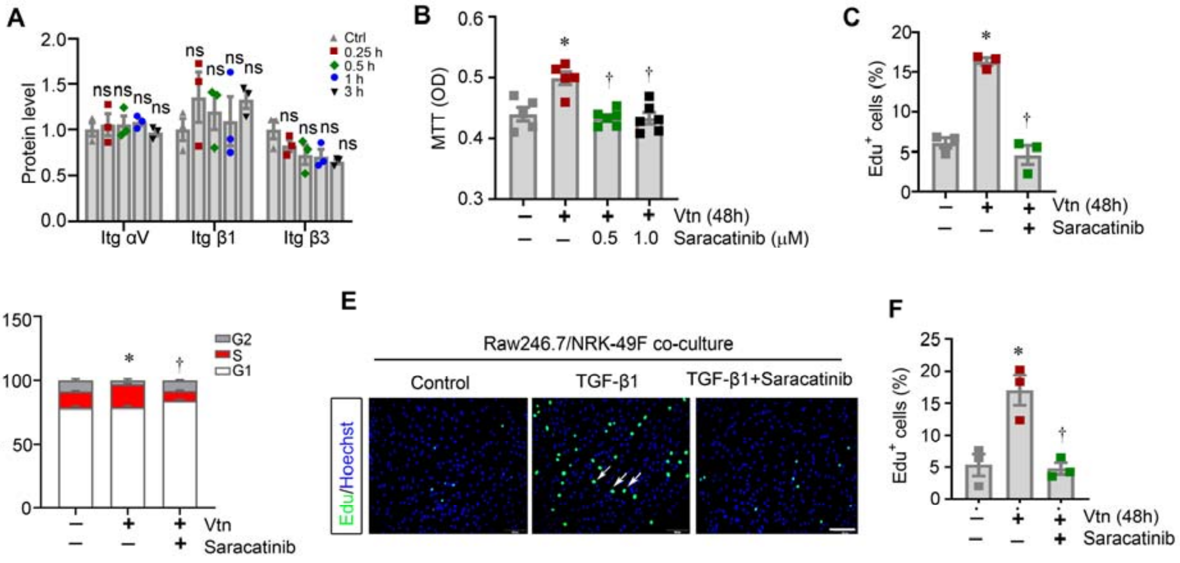

Supplementary Figure S2. Pharmacologic blockade of Src signaling inhibits fibroblast proliferation. (A) Quantitative data of Western blots show integrin αv, β1 and β3 expression in NRK-49F cells after treatment with human recombinant Vtn for different periods of time as indicated. n.s, non-significant (n = 3). (B) NRK-49F cells were incubated with Vtn at 50 ng/ml in the absence or presence of Src inhibitor Saracatinib for 48 h, and cell proliferation was assessed by MTT assay. $^{*}P < 0.05$ vs. control cells, $^{\dagger}P < 0.05$ vs Vtn alone (n = 4). (C) Quantitative data of EdU staining show that inhibition of Src signaling abolished Vtn-induced NRK-49F cell proliferation. $^{*}P < 0.05$ vs control cells, $^{\dagger}P < 0.05$ vs Vtn alone (n = 3). (D) Cell cycle distribution of NRK-49F cells in control, Vtn alone and Vtn plus Saracatinib group. $^{*}P < 0.05$ vs control cells, $^{\dagger}P < 0.05$ vs Vtn alone (n = 3). (E, F) Effect on the proliferation of fibroblasts co-cultured with Raw264.7 cells (E). (F) Quantitative data of EdU-positive cells in NRK-49F cells after co-cultured with Raw264.7cells. Arrowhead points to EdU-positive cells. $^{*}P < 0.05$ vs controls, $^{\dagger}P < 0.05$ vs TGF-β1 alone (n = 3). Scale bar, 100 μm.

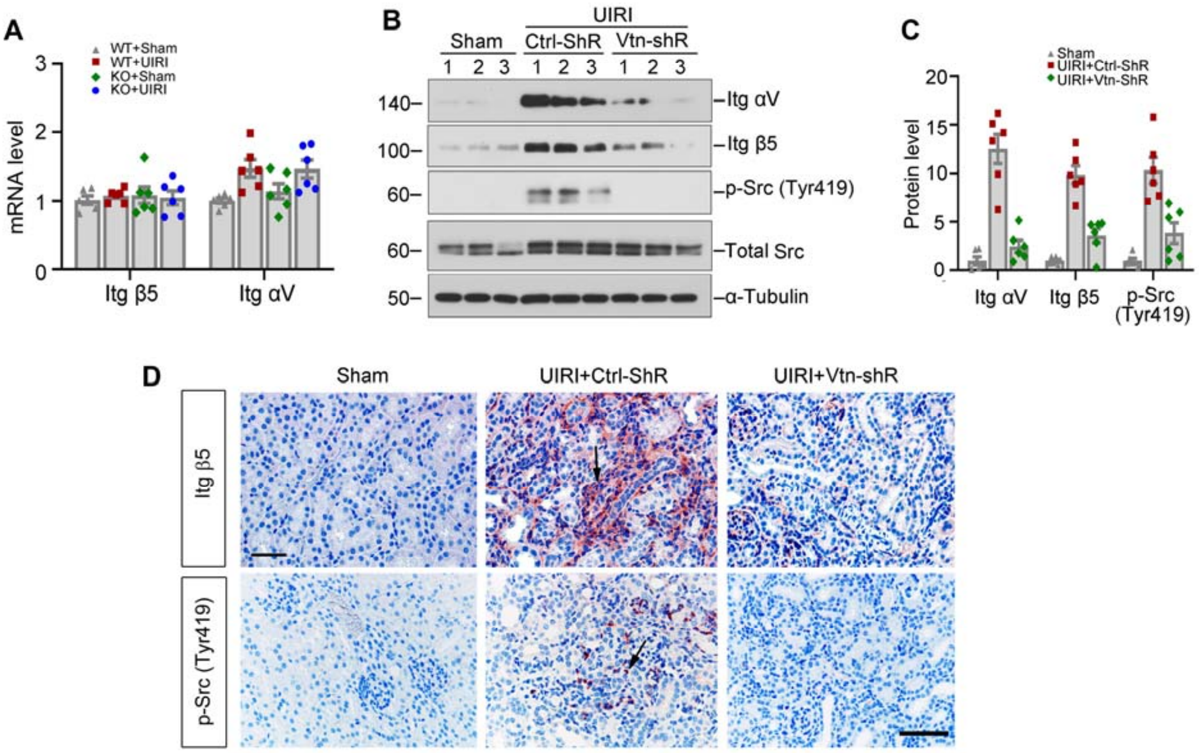

Supplementary Figure S3. Knockdown of Vtn inhibits integrin αvβ5/Src signaling in UIRI model. (A) Integrin αvβ5 mRNA expression in the kidneys of various groups of mice as indicated. WT, wild-type; KO, knockout mice. n.s, not significant (n =6). (B, C) Representative images of protein expression level (B) and densitometric quantification (C) show integrin αv, integrin β5, p-Src (Tyr419) and total Src expression. (D) Representative immunohistochemical staining of integrin β5 and p-Src (Tyr419) in different groups. Arrowheads point to positive area. $^{*}P < 0.05$ vs. Sham group, $^{\dagger}P < 0.05$ vs. UIRI plus Ctrl-ShR group (n = 6). Scale bar, 50 μm.

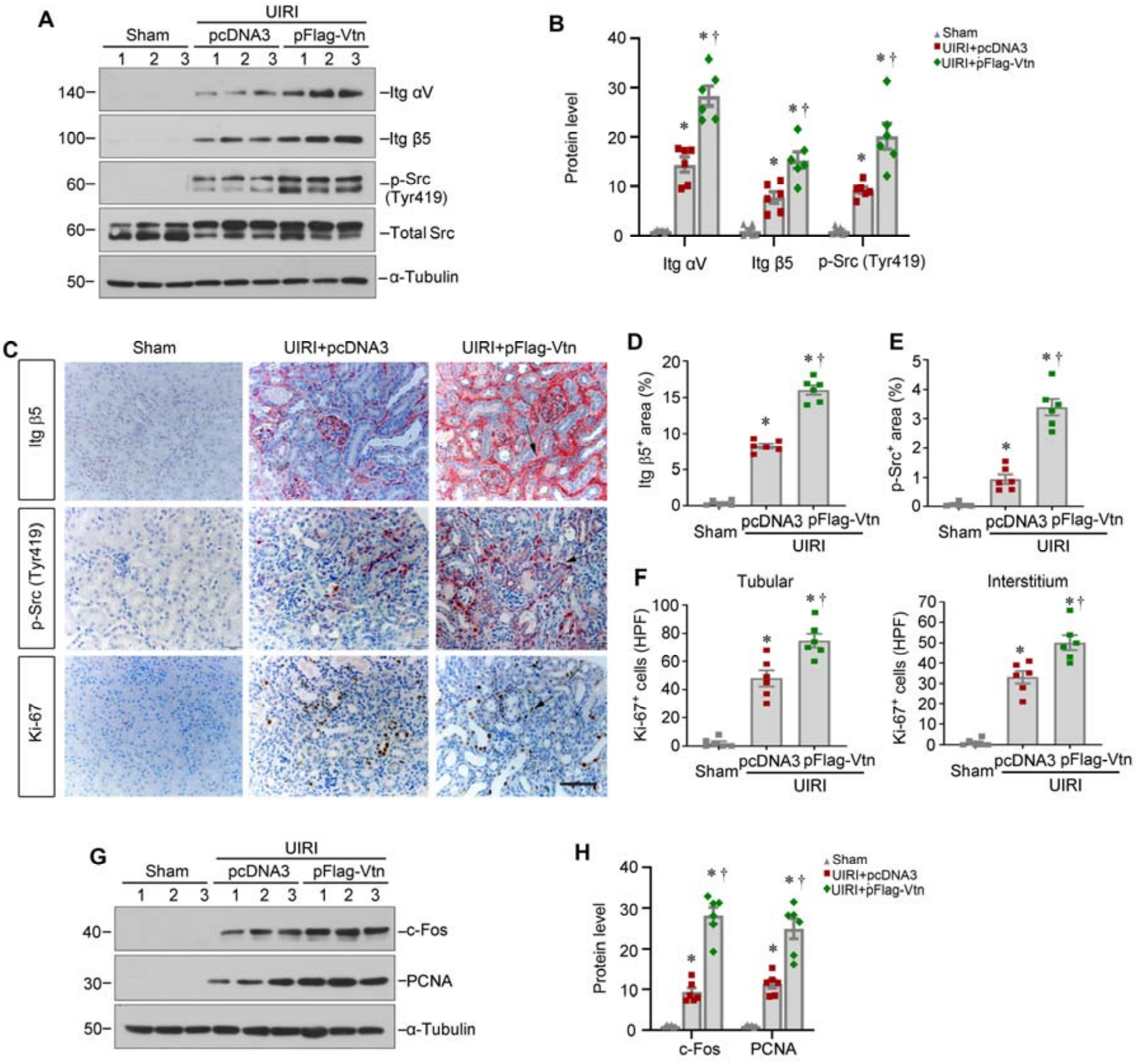

Supplementary Figure S4. Overexpression of Vtn activates integrin αvβ5/Src signaling in the UIRI model. (A, B) Representative images of protein bands (A) and densitometric quantification (B) show integrin αv, integrin β5, p-Src (Tyr419) and total Src expression. (C) Representative immunohistochemical staining of integrin β5, p-Src (Tyr419) and Ki-67. Arrowheads indicate positive area or cells. (D–F) Quantification of immunohistochemical staining results of integrin β5 (D), p-Src (Tyr419) (E), and Ki-67 (F). (G, H) Representative images of protein bands (G) and relative protein band density (H) show c-Fos and PCNA expression. $^{*}P < 0.05$ vs Sham group, $^{\dagger}P < 0.05$ vs UIRI plus PCDNA3 group (n = 6). Scale bar, 50 μm.

Supplementary Table S1. Demographic and clinical data of healthy subjects and CKD patients.

Characteristics	Cohort	
	Controls	CKD patients
**Gender-No.(%)**		
Male	6 (46)	59 (51)
Female	7 (54)	57 (49)
**Age at entry-years**		
Mean±SEM	39.31±3.50	44.73±2.12
Range	35-48	15-72
**eGFR(ml/min/1.73m2)-No.(%)**		
＞90	13 (100)	35 (30)
＜90	0 (0)	81 (70)
**Pathological Diagnosis-No.(%)***		
MN	0 (0)	26 (22)
DN	0 (0)	38 (33)
LN	0 (0)	10 (9)
IgAN	0 (0)	42 (36)
RCC	13 (100)	0 (0)

*MN, membranous nephritis; DN, diabetic nephropathy; LN, lupus nephritis; IgAN, IgA nephropathy; RCC, renal cell carcinoma.

Supplementary Table S2. The sources of antibodies used in this study.

Supplementary Table S2. The sources of antibodies used in this study			
**Antibodies**	**Catalogue number**	**Company**	**Location**
Rabbit monoclonal anti-Vitronectin (For WB/IHC)	NBP2-67511	Novus	Centennial, CO
Rabbit polyclonal anti-Vitronectin (For IP)	ab235987	Abcam	Cambridge, MA
Rabbit polyclonal anti-fibronectin	F3648	Sigma-Aldrich	St. Louis, MO
Mouse monoclonal anti-α-SMA	A5228	Sigma-Aldrich	St. Louis, MO
Rabbit monoclonal anti-Tenascin C	ab108930	Abcam	Cambridge, MA
Rabbit polyclonal anti-COL1A1	BA0325	Boster Biological Technolo[illegible]	Wuhan, China
Mouse monoclonal anti-PCNA	2586S	Cell Signaling Technology	Danvers, MA
Rabbit polyclonal anti-c-fos	BA0207-2	Boster Biological Technolo[illegible]	Wuhan, China
Rabbit monoclonal anti-c-Myc	5605S	Cell Signaling Technology	Danvers, MA
Rabbit monoclonal anti-Integrin αV	ab179475	Abcam	Cambridge, MA
Rabbit monoclonal anti-Integrin β5 (For WB/IHC)	ab184312	Abcam	Cambridge, MA
Rabbit polyclonal anti-Integrin β1	ab183666	Abcam	Cambridge, MA
Rabbit monoclonal anti-Integrin β3	ab210515	Abcam	Cambridge, MA
Rabbit monoclonal anti-Integrin β5 (For IP)	3629S	Cell Signaling Technology	Danvers, MA
Rabbit monoclonal anti-Phospho src(Y419)	ET1609-15	HUABIO	Boston, MA
Rabbit monoclona anti-Src	ab109381	Abcam	Cambridge, MA
Rabbit monoclonal to CD68	ab283654	Abcam	Cambridge, MA
Rabbit monoclonal anti-Ki67	ab16667	Abcam	Cambridge, MA
Rabbit monoclonal anti-vimentin	5741S	Cell Signaling Technology	Danvers, MA
Mouse Endomucin Antibody	AF4666	R&D Systems	Minnesota, MN
E-Cadherin (24E10) Rabbit mAb	3195	Cell Signaling Technology	Danvers, MA
Mouse monoclonal to Mannose Receptor	ab8918	Abcam	Cambridge, MA
Rabbit polyclonal to iNOS	ab15323	Abcam	Cambridge, MA
Rabbit polyclonal to TGF beta 1	ab92486	Abcam	Cambridge, MA
Mouse monoclonal anti-GAPDH	RM2002	Ray Antibody Biotech	Beijing, China
Mouse monoclonal anti-α-Tubulin	RM2007	Ray Antibody Biotech	Beijing, China
